# The microbiome’s fiber degradation profile and its relationship with the host diet

**DOI:** 10.1186/s12915-022-01461-6

**Published:** 2022-12-05

**Authors:** Yotam Cohen, Elhanan Borenstein

**Affiliations:** 1 The Blavatnik School of Computer Science, Tel Aviv University, Tel Aviv, Israel; 2 Sackler Faculty of Medicine, Tel Aviv University, Tel Aviv, Israel; 3 Santa Fe Institute, Santa Fe, NM USA

**Keywords:** Microbiome, Dietary fibers, Personalized nutrition, Diet

## Abstract

**Background:**

The relationship between the gut microbiome and diet has been the focus of numerous recent studies. Such studies aim to characterize the impact of diet on the composition of the microbiome, as well as the microbiome’s ability to utilize various compounds in the diet and produce metabolites that may be beneficial for the host. Consumption of dietary fibers (DFs)—polysaccharides that cannot be broken down by the host’s endogenous enzymes and are degraded primarily by members of the microbiome—is known to have a profound effect on the microbiome. Yet, a comprehensive characterization of microbiome compositional and functional shifts in response to the consumption of specific DFs is still lacking.

**Results:**

Here, we introduce a computational framework, coupling metagenomic sequencing with careful annotation of polysaccharide degrading enzymes and DF structures, for inferring the metabolic ability of a given microbiome sample to utilize a broad catalog of DFs. We demonstrate that the *inferred fiber degradation profile* (IFDP) generated by our framework accurately reflects the dietary habits of various hosts across four independent datasets. We further demonstrate that IFDPs are more tightly linked to the host diet than commonly used taxonomic and functional microbiome-based profiles. Finally, applying our framework to a set of ~700 metagenomes that represents large human population cohorts from 9 different countries, we highlight intriguing global patterns linking DF consumption habits with microbiome capacities.

**Conclusions:**

Combined, our findings serve as a proof-of-concept for the use of DF-specific analysis for providing important complementary information for better understanding the relationship between dietary habits and the gut microbiome.

**Supplementary Information:**

The online version contains supplementary material available at 10.1186/s12915-022-01461-6.

## Background

The gut microbiome, the ensemble of microorganisms that inhabits the gastrointestinal tract, has a tight relationship with various dietary habits and with the consumption of a variety of food items [1–3]. Variation in the host diet, and even changes in dietary regimes, is known to affect the composition of the gut microbiome by modulating the availability of essential nutrients that selectively promote the growth of specific commensal bacteria [1, 2, 4–6]. On the other hand, gut bacteria can degrade and ferment consumed nutrients and produce important secondary metabolites (most notably, short-chain fatty acids (SCFA)) that benefit the host [7–11]. Indeed, many studies have shown that certain bacteria in the gut can ferment food items, such as dietary fibers (DFs); grow; and produce useful SCFA both in vitro and in mice models [12–17]. Yet, a comprehensive characterization of which bacteria can thrive in response to various diets, and which food items (and specifically which DFs) each bacteria can degrade, ferment, and utilize, is still lacking [3, 5].

DFs are polysaccharides, mostly of plant origin, that cannot be degraded by the endogenous human enzymes, but only by the organisms residing in our gut [4]. They are commonly divided into two broad classes: soluble and insoluble, with each class containing various types of DFs. DFs differ from one another in the main sugar component of which they are composed, the chemical bonds linking their main chain, the side chains they may include, the degree of polymorphism, and several additional attributes. As DFs can only be degraded by the microbiome and serve as an important nutrient source for microbiome members, specific shifts in bacterial population can be observed in response to consumption of distinct fibers in a variety of clinical trials [18–23]. Moreover, clinical studies have identified changes in the production of SCFA and in their beneficial contribution to specific disease states in response to DF supplementation [19, 24, 25]. Such effects and interactions with specific bacteria, however, were shown only for a handful of DFs, and a more comprehensive characterization of microbiome compositional changes in response to different DFs consumption is not yet available [26, 27].

To address this challenge and to systematically map the relationship between various microbial species and DFs, several attempts have been made to elucidate the functional capacity of the microbiome in respect to polysaccharide degradation. These efforts predominantly focused on annotation of gene families involved in glycoside hydrolysis (GH)—a process that breaks bonds by the insertion of a water molecule, and polysaccharide lyase (PL), which cleave complex carbohydrates using an elimination mechanism [4, 28–30]. While useful, the classification of GH and PL gene families is relatively broad and based primarily on sequence similarity, generally failing to link specific gene families with the degradation of specific polysaccharides. Additional efforts to model the functional ability of microbial species to utilize complex polysaccharides have further focused on identifying clusters of genes termed polysaccharide utilization loci (PUL) [31–33] in bacterial genomes. Such efforts aim to detect clusters of genes encoding GH and PL enzymes surrounded by genes encoding for proteins and enzymes such as transporters, phosphatases, esterases, membrane-bound binding proteins, and transcription factors, thus hinting toward a specific complex polysaccharide degradation capacity. Yet, despite these extensive studies, the ability to pinpoint and, more importantly, to quantify the ability to degrade specific DFs remains elusive. Moreover, the approaches above are suitable primarily for genomic-based studies and are limited in their ability to generate meaningful insights directly from metagenomics data or to infer the DF degradation capacity of mixed bacterial communities.

An earlier, and the most successful to date, attempt to address this challenge was able to offer an outlook into the capability of each genome to degrade glycans by constructing a computational pipeline based on manual annotation of GH and PL enzymes and the set of specific bonds they could break [34]. This impressive framework, termed GlyDeR, then predicted the ability of each bacterial species to degrade glycans, distinguishing, for example, between plant-based and animal-based glycans. Yet, while some analysis of whole-shotgun metagenomic samples was performed, GlyDer focused primarily on the genomic capacity of bacterial species to degrade glycans and on relatively broad glycan classifications, rather than on comprehensive quantification of the ability of a given metagenome to degrade specific DFs and to link such abilities to specific dietary habits.

Here, inspired by GlyDeR and utilizing some of the approaches it introduced, we present a simple yet powerful framework for inferring the metabolic potential of a given microbiome to degrade a large set of DFs, by coupling metagenomics sequencing with careful annotation of DFs’ chemical structures and polysaccharide degrading enzymes. We validate the obtained “inferred fiber degradation profiles” by demonstrating their associations with various food items consumed by the host and their distinct fiber content, utilizing multiple metagenomic cohorts. Furthermore, we show that such fiber degradation profiles are more strongly linked to the host’s dietary habits than taxonomic or functional profiles. Finally, we demonstrate that our framework can be applied to large metagenomics cohorts, revealing novel insights about these cohorts and detecting shared metabolic patterns among distinct populations with similar DF consumption habits.

## Results

### Inferring fiber degradation profile from metagenomic samples

In this study, we focus on the capacity of different microbiomes to degrade DFs, directly inferring this capacity from shotgun metagenomic data. Extending previous approaches [34], we specifically aim to couple metagenomic data with carefully annotated enzyme, fiber, and protein entities, ultimately calculating an *inferred fiber degradation profile* (IFDP) for each metagenomic sample. We wish to show that this analytical framework can provide accurate information into the differences and similarities in fiber degradation capacities across microbiome samples and provide intriguing insights into the relationship between the microbiome fiber degradation capacity and the host’s diet.

**Fig. 1:**
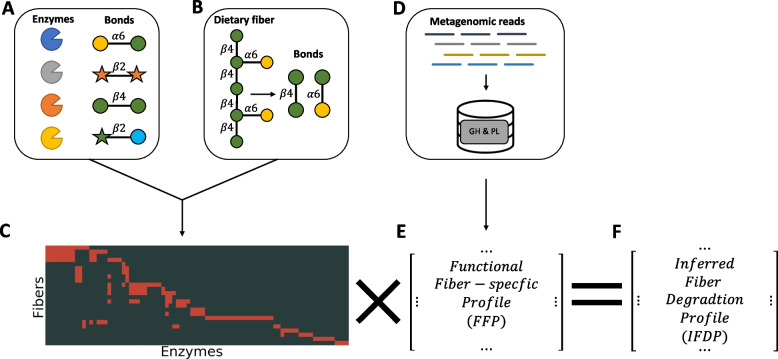
A schematic illustration of our framework. **A** Annotation of GH and PL enzymes, indicating the glycoside bonds each enzyme can break down. **B** Annotation of dietary fibers, indicating the set of glycoside bonds each enzyme includes. **C** A matrix representation of the links between dietary fibers and GH/PL enzymes. A red cell indicates that the enzyme in this column is capable of operating on one of the bonds included in the dietary fiber in this row. **D**, **E** Mapping of metagenomic reads to a database of GH and PL sequences, resulting in a matrix describing the abundance of each GH/PL enzyme in each sample. This profile is further referred to in our work as the functional fiber-specific profile (FFP). **F** The inferred fiber degradation profile (IFDP) can be obtained by multiplying the DF-enzyme matrix by the FFP

To calculate the IFDP, we first constructed a catalog of all the enzymes that have the capacity to catalyze the degradation of polysaccharides, including primarily all glycoside hydrolysis (GH) and polysaccharide lyase (PL) enzymes, based on enzyme commission numbers (see the “Methods” section). We chose to build this database using the UniProt database, rather than the Carbohydrate-Active Enzymes (CAZy) database [31] (which represents a vast source of knowledge on hydrolysis enzymes), since CAZy often categorizes enzyme entries as broad non-fiber-specific GH and PL families, thus lacking a specific chemical reaction which is crucial for our analysis. We then generated and manually curated a dataset of fiber-enzyme interactions by first cataloging the chemical bonds that each GH and PL enzyme can break (relying largely on a previously introduced annotation system [34]) and then cataloging the set of chemical bonds included in each dietary fiber (Fig. 1A, B, Additional files 1 and 2). Combining these two catalogs, we constructed an enzyme-fiber interaction matrix, in which each entry *M*_*ij*_ denotes whether enzyme *i* has the capacity to break down a bond present in dietary fiber *j* (Fig. 1C). We additionally downloaded all GH and PL protein sequences from the UniProt database [35], using both Swiss-prot (manually annotated and reviewed sequences) and Trembl (automatically annotated sequences), and used these sequences to build a reference database of all fiber-degrading enzyme sequences (Fig. 1D). Additional information can be found in the “Methods” section.

Given a metagenomic sample, we then carefully mapped all shotgun reads to the reference protein database described above to obtain the abundance of each enzymatic gene of interest. We refer to these abundances as the *functional fiber-specific profile* (FFP) of the metagenome, describing the set and abundances of fiber-degrading enzymes in the community (Fig. 1E). Finally, we multiplied the obtained FFP by the enzyme-fiber interaction matrix, to obtain the IFDP, describing the relative capacity of the metagenome to degrade each DF (Fig. 1F).

While this framework is aimed to be primarily applied to metagenomic data, as a simple test case for its construction and accuracy of our annotation process, we first applied it to a large set (>1000) of *Prevotella copri* genomes (see the “Methods” section). Specifically, we used our framework to calculate the IFDP of each genome, clustered the genomes based solely on their IFDP, and compared the resulting clusters to those previously reported based on a set of 400 universal marker genes [36]. As seen in Fig. S1A, IFDP-based clusters successfully mirror the four distinct marker gene-based clades, highlighting our framework’s ability to accurately capture genomic metabolic properties. Moreover, we compared this IFDP-based clustering to the clustering obtained using the FFP (GH/PL enzyme-based) profile, by applying a principal component analysis to the *P. copri* genomes using each of these two representations. We found that the various clusters are easily noticeable in both representations (with IFDP in fact explaining more of the variance; Fig. S1B-C) and that the first principal component strongly correlates across the two representations (Spearman *r*=0.64, *p*<1e−125; Fig. S1D). These findings suggest that while the IFDP summarizes mostly the metabolic potential to degrade a specific set of fibers, its ability to distinguish these genomes is comparable to a representation based on GH/PL enzyme distribution.

Finally, to provide a more rigorous, experimentally based validation, we have obtained data from an in vitro experiment study [37], where multiple *Prevotella copri* strains that represent the clusters above were isolated from human donors and grown on various plants derived dietary fibers. Comparing the predicted IFDP of each cluster to these experiments, we found overall good agreement. For example, the IFDP of cluster B genomes suggests that they lack the ability to degrade arabinoxylan, arabinan, and glucomannan (Fig. S1A), and indeed strains from this cluster were not able to grow on these dietary fibers as a sole carbon source. Similarly, strains from clade C could not degrade beta-glucan, xyloglucan, and glucomannan, and the strain representing clade D was not able to grow on fructans (i.e., inulin and levan), as also suggested by the IFDP of the genomes from these clades. Strains from clade A presented a range of degradation capacities in the experiment, as reflected also by their IFDP profiles. We also noticed some discrepancies; for example, strains from cluster C were able to degrade rhamnogalacturonan, which was not predicted by our profile. In total, out of the 15 cases in which the in vitro assay suggested that a given clade cannot grow on a certain fiber, in 8 cases, the clade had the lowest IFDP score for that fiber, and in 4, the second lowest score (12 cases overall; *p*<0.0005, permutation-based test; see the “Methods” section). Similarly, out of the 16 cases in which the in vitro assay suggested that a given clade can grow well on a certain fiber, in 7, the clade had the highest IFDP score for that fiber, and in 5, the second highest score (12 cases overall; *p*<0.035, permutation-based test).

Overall, however, these experimental results provide further validation for the ability of the IFDP to capture functional properties concerning dietary fiber degradation.

### IFDP is tightly linked to the host diet

Given the framework described above, we set out to investigate the relationship between the IFDP and the host diet. We hypothesize that the degradation capacity (as quantified by the IFDP) of DFs commonly consumed by a given group of hosts will be higher compared to hosts who more rarely or never consume these DFs. To test this hypothesis, we analyzed data from four different metagenomics-based studies, each characterizing microbiome samples from different groups of hosts with distinct fiber consumption regime. The first study examined the microbiomes of mouse pups whose mothers were fed one of several distinct diets [38]. The second study examined the microbiome of a diverse group of non-human primates, specifically focusing on the potential difference between folivores and non-folivores [39]. The last two studies examined the microbiome of specific human populations: one characterized the microbiome of the Hadza tribe across different seasons (and accordingly different dietary regimes) [40], and the other focused on the microbiome of two distinct Peruvian tribes with different dietary preferences. We used the framework above to calculate the IFDP profile of each sample in these datasets and compared the obtained IFDP from the various groups in each study and their relationship to known information about dietary differences between groups.

**Fig. 2:**
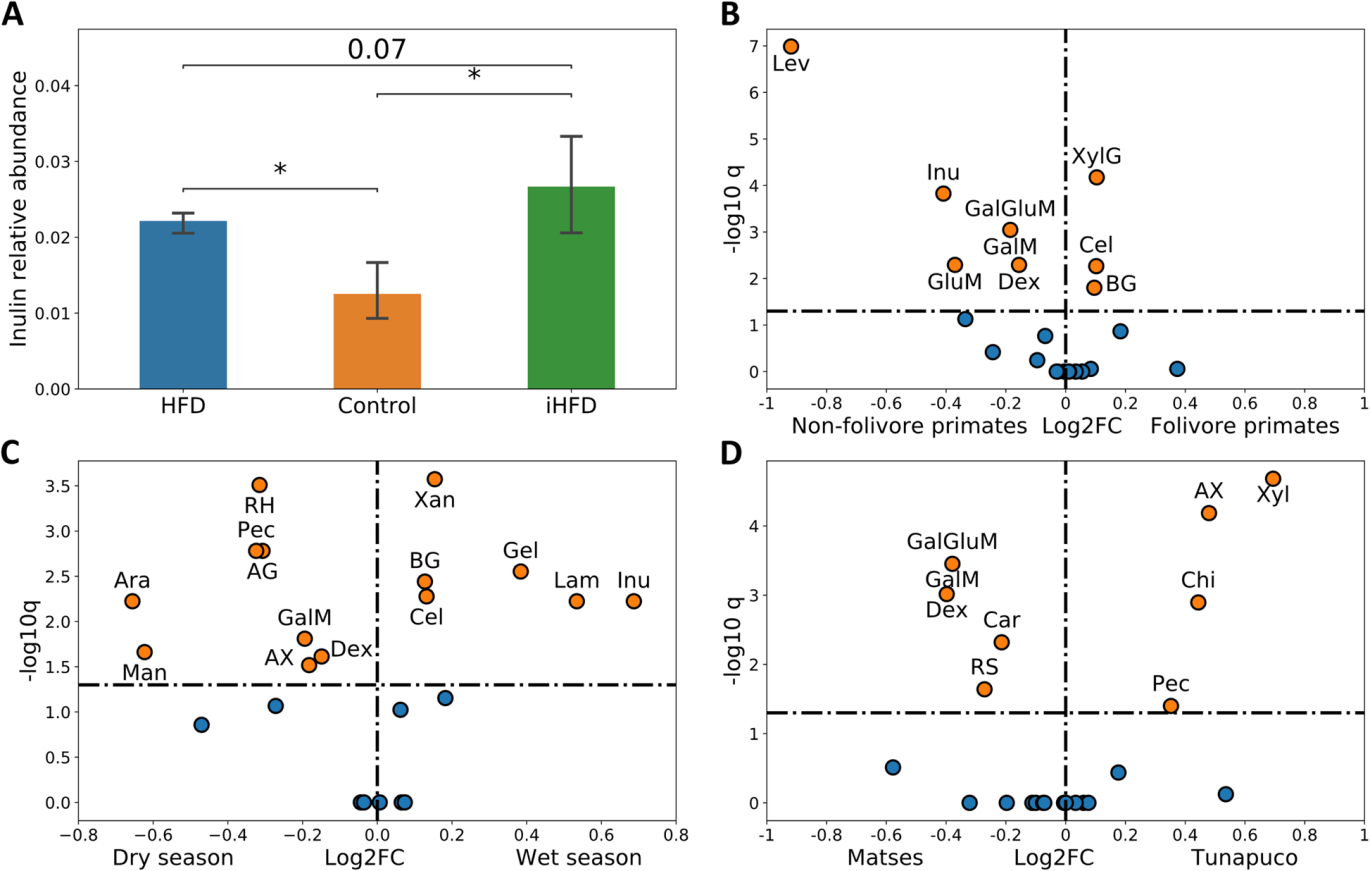
Differences in the degradation capacity of various DFs, as indicated by the IFDP, across populations with different dietary habits. **A** A bar plot describing inulin degradation capacity (as indicated by the IFDP) in mice from different diet groups (the asterisk symbol indicates *p*<0.05). **B**–**D** Volcano plots describing differences in degradation capacity of different DFs between **B** folivore and non-folivore primates, **C** Hadza tribe population in dry and wet seasons, and **D** two distinct Peruvian tribes. The *X*-axis represents the log fold change of the fiber degradation ability. *p* values are corrected for multiple hypotheses. The dashed line at ~−1.3 *y*-axis (−log_10_ of the *p* value) denotes the 0.05 *p* value significance threshold. Dietary fiber abbreviations: Inu, inulin; Lev, levan; BG, beta-glucan; Cel, cellulose; Xyl, xylan; GluM, glucomannan; GalM, galactomannan; Man, mannan; AX, arabinoxylan; Gal, galactan; Ara, arabinan; AG, arabinogalactan; Dex, dextran; Chi, chitin; RH, rhamnogalacturonan; Pec, pectin; Car, carrageenan; GalGluM, galactoglucomannan; Alg, alginate; Xan, xanthan; Xyl, xloglucan; Lam, laminaran; Gel, gellan; RS, resistant starch

Focusing initially on the mouse dataset allowed us to examine our hypothesis in a simple, well-characterized, and well-controlled setting. In this study, mothers were partitioned into three groups, each fed a different diet: the first group was fed a control diet consisting of 10% fat, 20% protein, and 70% carbohydrate; the second was fed a high-fat diet (HFD) with 45% fat, 20% protein, and 35% carbohydrate; and the third was fed a high-fat diet with supplementation of 10% inulin (iHFD). The microbiome of the offspring (*n*=15, five per group) was sampled at weaning. Our hypothesis therefore predicts that the microbiomes of mice whose mothers were fed inulin would exhibit an increased inulin degradation capacity. Indeed, examining the calculated IFDP for each sample, we found a significantly higher inulin degradation capacity in the iHFD group compared to the control group (*p*<0.05, Mann-Whitney test; Fig. 2A). A similarly increased capacity was also observed in comparison to the HFD group, but did not reach our significance threshold (*p*=0.07), potentially due to the small sample size.

To test our hypothesis in a more systematic manner and in more complex and natural settings, we turned to the primate dataset. This dataset describes the gut microbiomes of 89 non-human primates, spanning 18 primates’ species, of which 44 are folivores (i.e., whose diet is composed primarily of leaves) and 45 are non-folivores (whose diet includes mostly fruits and insects). Focusing on the microbiomes of folivores vs. non-folivores, our hypothesis would accordingly predict that the calculated IFDP of folivores will indicate an increased capacity to degrade cellulose and xyloglucan—the two main structural components of the primary cell wall of leaves [41, 42], compared to the IFDP of non-folivores. Indeed, comparing the calculated IFDPs across samples in this dataset clearly demonstrated this pattern, with a higher capacity to degrade these two main structural leaf components (as well as beta-glucan) in the IFDP of folivores (*p*<0.05, Mann-Whitney test; Figs. 2B and S2A-B). Moreover, this analysis has also demonstrated that the capacity to utilize a variety of other fibers, especially the fructans, inulin and levan (Figs. 2B and S2C-D), as well as glucomannan, galactomannan, and dextran, was higher in the IFDP of non-folivores, potentially reflecting an adaptation of the microbiome to a more diverse diet with a higher variety of fibers.

The third dataset, obtained from the Hadza population in Tanzania (*n*=35), contains samples collected in two seasons, the dry season and the wet season, with samples from the dry season collected in two consecutive years. Importantly, the Hadza diet is based on available resources in their environment and includes five main components: tubers, berries, meat, baobab, and honey [43]. While tubers and honey are available all year long, meat and baobab fruits are consumed more frequently during the dry season, whereas berries are consumed mostly during the wet season [40, 44]. Notably, the fiber content of baobab fruits differs considerably from that of berries, with pectin constituting the large portion of the baobab fruits’ fiber content [45] and fructans (fructose-containing DFs such as inulin and levan) constituting a large portion of berries’ fiber content [40]. The calculated IFDPs clearly mirrored this pattern, demonstrating a higher capacity to ferment and ultimately break down pectin in the dry season and inulin in the wet season (Fig. 2C). Furthermore, the IFDP suggested that the gut microbiome’s ability to utilize pectin and additional arabinose containing fibers (such as rhamnogalacturonan and arabinan) were noticeably higher in the dry season, possibly due to the effect of pectin on changes in the enzymatic ability to break arabinose bonds. To further confirm that the capacity to degrade inulin and pectin is indeed distinctly different between dry and wet seasons, we repeated the analysis treating the dry season cohorts of each year separately (also avoiding biases of different group sizes). Confirming our hypothesis, we again found that the ability to degrade inulin was significantly higher in the wet season compared to *either* of the two dry seasons (Fig. S2E), while the ability to utilize pectin, arabinan, and rhamnogalacturonan was significantly lower in the wet season compared to either of the two dry seasons (Fig. S2F-H).

Finally, we examined the fourth dataset, obtained from a study on the differences between urban and industrialized microbiome profiles, using samples from the Peruvian tribes Matses and Tunapuco (*n*=36) [46]. These two tribes have substantially different diets, with Matses primarily consuming cassava, plantain, and fish as their main nutritional source, whereas Tunapuco diet is based primarily on potatoes, corn, tubers, rice, a wide variety of fruits, and meat [46]. Examining the IFDPs calculated for the different samples in this dataset, we observed a few significant and intriguing patterns (Fig. 2D). First, the abilities to breakdown the bonds in arabinoxylan and xylan were both significantly higher in the IFDP of individuals from the Tunapuco tribe (see also Fig. S2I-J). This is in agreement with the high consumption of rice and corn, both of which are rich sources of arabinoxylan [47]. In addition, an increased capacity to utilize pectin was observed in this tribe, potentially reflecting the consumption of a variety of fruits such as oranges, apples, and mangos that have high concentrations of pectin (Fig. S2K). In the Matses tribe, the capacity to utilize resistant starch and carrageenan, a marine dietary fiber, was significantly higher, again successfully mirroring this tribe’s dietary consumption of plantain and fish (Fig. S2L-M).

Combined, these findings highlight the extremely tight link between the host’s diet and the functional capabilities of its microbiome in terms of the microbiome’s ability to degrade and utilize energy encompassed in dietary fibers. Notably, this link was evident in all the datasets we analyzed above, including both links that reflect the impact of short-term dietary regimes (such as in the mice and Hadza studies) and links that mirror the life-long dietary habits (such as in the primate and Peruvian datasets).

### IFDP reflects the host diet better than other microbiome-based profiles

Given the strong association demonstrated above between the host’s diet and IFDP, we set out to compare this association with those that may exist between the host’s diet and more commonly used microbiome-based taxonomic and functional profiles. Specifically, we analyzed samples from both the primates and the Hadza datasets described above, this time, quantifying the taxonomic profile of each sample using metaphlan2. We examined the obtained taxonomic profiles both at the genus level (referring to these as *taxonomic genus profiles* or TGP) and at the species level (referring to these as *taxonomic species profiles* or TSP). We additionally examined functional profiles for each sample, including its *functional fiber-specific profile* (FFP) describe above, as well as its *functional complete profile* (FCP), describing the complete set of functional capacities (i.e., not only those related to DF degradation as in the FFP), obtained by mapping reads in the sample to all bacterial proteins in UniProt (see the “Methods” section).

We first examined the relationships of the various profiles, to determine whether different profiles capture the same information about a given sample. Since each profile has a different dimension, we used a principal component analysis of the primates dataset and examined the correlation of the first principal component across profiles. This analysis suggested that the IFDP is relatively unique and shares little of the explained variance with other profiles (Fig. S3). Below, we further compare the association between diet and each of these profiles to the link between diet and IFDP.

**Fig. 3:**
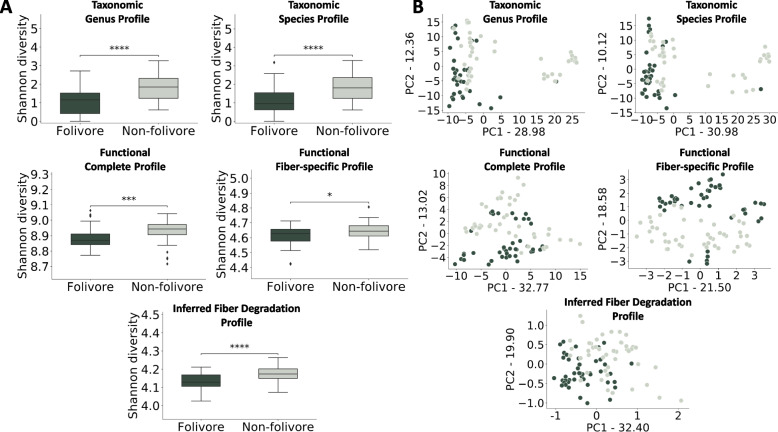
Comparing the link of the IFDP and of other microbiome-based profiles to the host diet. **A** Box plots comparing the Shannon diversity index between folivore and non-folivore primates of various microbiome-based profiles. *p<0.05, **p<0.01, ***p<0.001, ****p<0.0001. **B** Principal component analysis (PCA) plots, colored by folivore vs non-folivore primates, based on various microbiome-based profiles

Focusing on the primate dataset, we examined the Shannon alpha diversity index for each profile, evaluating potential differences between folivores and non-folivores. Perhaps not surprisingly, folivore primates exhibited significantly lower diversity compared to non-folivores across all microbiome profiles, including the IFDP, likely reflecting the limited diversity of their diets (Fig. 3A). We then set out to examine the beta diversity between samples using a principal component analysis and applying a permutational multivariate analysis of variance (PERMANOVA; see the “Methods” section) to assess how well each profile distinguishes between folivores and non-folivores (Fig. 3B). This analysis clearly demonstrated that IFDP separated the folivore and non-folivore diet groups better than all other profiles (PERMANOVA *p* = 0.016, 0.037. 0.08, 0.02, and 0.001 for TGP, TSP, FCP, FFP, and IFDP, respectively). Since animal phylogeny class has been shown to have a higher contribution and a stronger impact on shaping the gut microbiome than diet [39] (specifically in the dataset we are using here), we decided to control for the phylogeny class in our analysis. Notably, IFDP remained the profile that best separated folivore and non-folivore primates (and the only one that remained significant) even when controlling for the phylogenetic class of the various primates (PERMANOVA *p* = 0.26, 0.44, 0.24, 0.1, and 0.014 for TGP, TSP, FCP, FFP, and IFDP, respectively; see the “Methods” section). Overall, these findings highlight the ability of the IFDP to detect the effect of diet on gut microbiome features, despite the stronger phylogeny signal.

Interestingly, extending our alpha diversity analysis to the Peruvian datasets, and comparing the alpha diversity of the two tribes described above to a non-rural group obtained from the same study (for which detailed fiber consumption data are lacking), we find that the Matses tribe exhibits significantly decreased FFP and FCP diversity compared to the other groups but comparable IFDP diversity (Fig. S4A-C). This may suggest that the restrictive consumption of food items by the rural Matses tribe leads to specific enzymes from specific bacteria to be more dominant in the microbiome, yet their fiber degradation profile, while unique, has comparable diversity to the other groups.

**Fig. 4:**
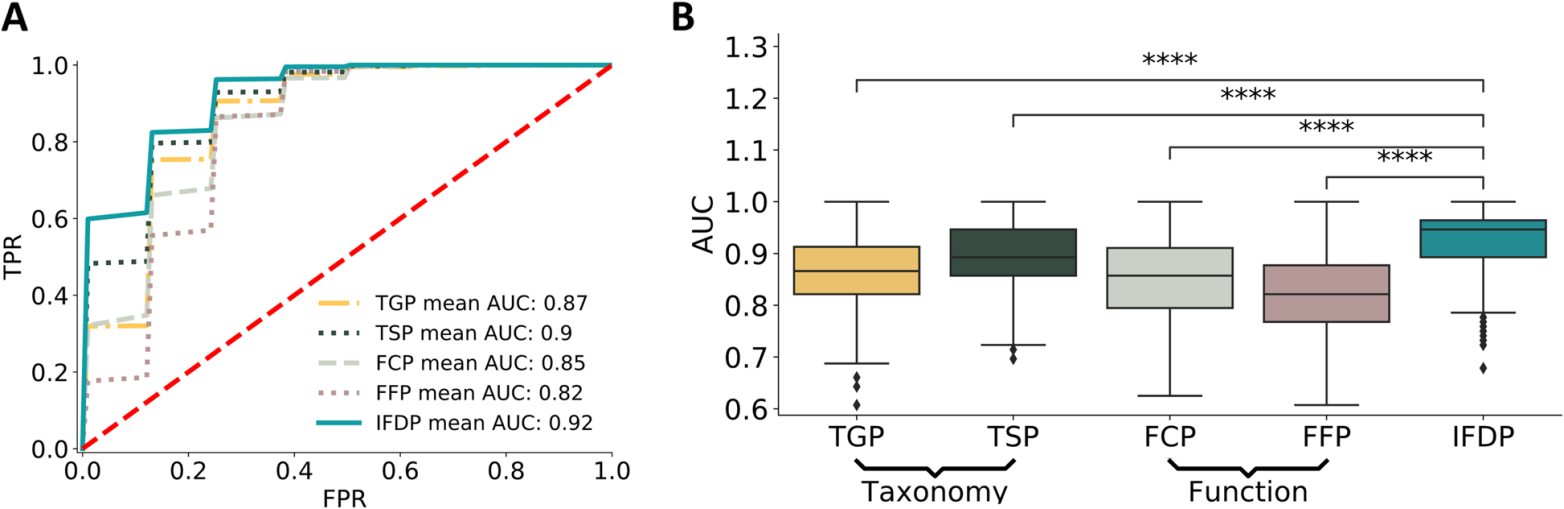
Predicting wet vs. dry season in the Hadza dataset using various microbiome-based profiles. **A** ROC curves describing five random forest classifier performances. **B** Box plot of the ROC AUC score obtained using each microbiome-based profile across all cross-validation iterations in each predictor. ****p<0.0001

Given the information captured by the IFDP about the host diet in the primate dataset, we next examined whether similar information may be captured in human IFDPs and whether this information can be used to infer the host dietary habits more accurately than other microbiome-based profiles. To this end, we again analyzed the Hadza dataset, calculating the five different profiles described above (namely, TGP, TSP, FCP, FFP, and IFDP) for each sample. We then tested how accurately we can predict (using a random forest classification with cross-validation) whether a sample was collected in the dry or wet season, based on each of these profiles (see the “Methods” section). We found that predictor models based on IFDP outperformed predictors based on any other profile (Fig. 4A, B). Specifically, the IFDP-based predictor achieved the highest mean ROC AUC score (0.92) compared to taxonomy- and function-based predictors (mean ROC AUC 0.87, 0.9, 0.85, and 0.82 for TGP, TSP, FCP, and FFP, respectively).

These results convincingly show that the IFDP can serve as a key marker for the host diet, providing important and powerful information regarding the host microbiome functional abilities in relation to dietary habits.

### Exploring the IFDP in large-scale metagenomic cohorts

Following our analysis above and the tight link between IFDP and the host’s diet observed in well-characterized and carefully studied cohorts, we finally turned to explore the IFDP of microbiomes from very large cohorts of human metagenomes from across the globe. Our main focus was using our framework to discover global signatures of DF degradation capacities and relating them to unique dietary habits. To this end, we carefully collected ~700 human gut metagenomes, obtained from nine different countries and three different continents, including Austria, Netherlands, and Spain (Europe); Ghana, Ethiopia, and Tanzania (Africa); and China, Mongolia, and India (Asia). Importantly, while all samples were obtained from healthy individuals (as defined by each of the various studies), comprehensive metadata regarding these samples are not available, and it is reasonable to assume that multiple factors, including diet, but also lifestyle and other factors (e.g., industrialized vs. non-industrialized), contribute to variation in their microbiomes. With that in mind, the following analysis is primarily meant to serve as an intriguing and potentially speculative proof of concept, demonstrating that it may be possible to detect diet-related signals even without comprehensive knowledge about population-level samples. Specifically, we applied the pipeline above to each sample to quantify its IFDP, with several small adjustments to better control for differences between sample sets (see the “Methods” section). We hypothesized that similarities and differences in the cuisines of different countries may be reflected in the IFDPs calculated for samples obtained from these countries.

**Fig. 5:**
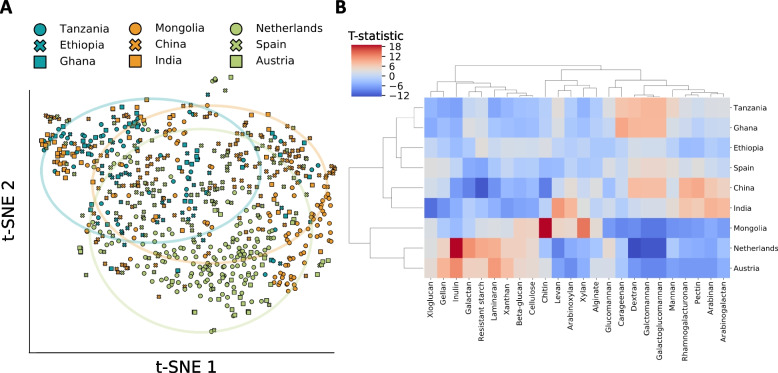
Comparing IFDPs across large-scale global metagenomic cohorts. **A** t-SNE plots based on the IFDPs of ~700 metagenomes from different countries colored and marked by continent and country. **B** Hierarchical clustering performed on a matrix containing t-statistics, describing the ability of each countries’ population (rows) to degrade a specific DF (columns) compared to all other populations. The color bar legend denotes the t-statistic of each country for each fiber

We examined the overall variation in IFDP, using t-SNE—a dimensionality reduction algorithm. This analysis demonstrated notable variation in the IFDPs of samples from different continents, with samples from the African cohorts, for example, clustering together and away from samples from European cohorts (Fig. 5A). Moreover, in agreement with the main food item consumed in each country [48–51], it could also be seen, for example, that samples from Austria clustered closer to samples from the Netherlands than to those from Spain.

Next, we concentrated our efforts on examining the relationships between the degradation capacities of specific DFs in certain countries and these fibers’ consumption habits in these counties. To this end, for each DF, we compared the DF degradation capacity in each country to its degradation capacity in all other countries (as measured by the IFDP) using a *t*-test (with Bonferroni correction). In total, we tested the 24 distinct dietary fibers for each of the nine countries, identifying on average 13.2 DFs for which each country exhibited significantly increased degradation capacities in comparison to other countries (Fig. 5B). Hierarchal clustering of the obtained t-statistic demonstrated, as above, substantial similarity between the IFDPs of African countries, as well as between the IFDPs of Austria and Netherlands, though interestingly, China and Mongolia exhibited marked differences in IFDPs for some DFs. Moreover, examining which fibers were associated with increased degradation capacities in different countries, further mirrored patterns in these countries’ cuisines. For example, both Austria and Netherlands had the highest degradation capacity for galactan, resistant starch, and inulin, potentially reflecting the relatively high consumption of dairy products and potatoes (rich sources of galactan and resistant starch) in these countries [48–51]. Similarly, the increased capacity to degrade arabinoxylan, which is present in all major cereal grains and most notably in rice [47], in China, Mongolia, and India, may reflect the use of rice as a staple food in these countries [52, 53]. And finally, the increased capacity to degrade pectin—a DF present in peel portion of citrus fruits [54], in China, India, and Spain, may be linked to these countries being among the leading citrus fruit-producing countries [55].

Unfortunately, since comprehensive dietary information and especially, information about specific fiber content in each diet, is challenging to obtain, we could not explain all patterns observed in these global data. These include, for example, the Mongolia cohort’s ability to degrade xylan and chitin, the India cohort’s high potential to utilize levan, and Tanzania and Ghana cohorts’ ability to utilize galactomannan, dextran, and carregenan. Overall, however, this exploratory analysis demonstrated again how our framework and the calculated IFDP can be used to obtain intriguing insights into the relationship between dietary habits and DF consumption and the functional capacity of the microbiome on a large scale, even when relatively little complementary metadata are available.

## Discussion

Overall, we presented a new outlook into the functional capacities of the gut microbiome, focusing on the microbiome’s ability to degrade different types of DFs and introducing a computational framework for extracting the microbiome’s fiber degradation profile directly from metagenomics data. We have further shown that this profile is closely linked with the dietary habits of the host, both across different host species and human cohorts. Moreover, we demonstrated that this calculated profile can be useful for inferring various properties concerning the host’s diet, beyond those that can be obtained from the commonly used functional and taxonomic microbiome profiles.

Notably, the unique perspective on the microbiome’s functional capacity facilitated by our framework provides information about the host diet across a wide range of environmental and physiological settings. This framework could accordingly serve as a complementary and valuable microbiome analysis technique in complex study designs, where multiple factors, both biological and technical, might influence the obtained microbiomes’ compositions. Furthermore, the IFDP could provide information about potential interactions between the host diet and microbial functional capacities, even when dietary information is poor or not available.

It is also worth noting that in contrast to taxonomic profiling, and to some extent even to standard functional profiling, the fiber degradation profile generated by our framework encapsulates relevant mechanistic information, for example, about the types of chemical bonds each enzyme can break and the types of bonds present in each DF. We believe that this mechanism-based approach is one of the strengths of our framework, and primarily, that it contributes greatly to the interpretability of our findings. It is, nonetheless, interesting to align changes in the IFDP with taxonomic shifts, studying, for example, how ecological shifts in the microbiome that occur in response to dietary interventions might be driven by (and in turn drive) changes in the microbiome capacity to utilize available fibers from the diet [56, 57]. Such analyses may allow us to test whether the increased ability to degrade dietary fibers by some bacteria might impact changes in microbiome composition. Indeed, our analysis above has demonstrated several potential cases of such functional plasticity, including, for example, seasonal changes in the microbiome of the Hadza tribe that follow the fiber content of available foods.

Importantly, this fiber-centric approach may also have intriguing translational applications. Specifically, DFs are known to offer many health benefits, some due to their physiological and functional properties, but others solely via the degradation of the various fibers by the microbiome. Our framework could serve as a key component in designing a tool for a personalized fiber-specific recommendation system, aiming to optimize DF-based health benefits in accordance with the host’s specific microbiome composition. Such applications, however, will likely require moving from the cohort to the individual level when analyzing IFDPs. Similarly, while many studies have demonstrated how prebiotics can modulate the microbiome, relatively few DF-based interventions have been clinically tested to carefully characterize their impact on the microbiome. Our framework can provide an estimation for the degree at which a dietary fiber could be utilized by the microbiome, highlighting the potential for microbiome modulation using specific DF interventions. Moreover, our approach can be used for predicting the effect size of such intervention studies, and combined with additional genomic data, might pinpoint the most appropriate intervention mechanism to directly modulate the microbiome composition in a specific direction.

Notably, however, our framework (and its current implementation) has several caveats. First, while our analysis has demonstrated that the IFDP is tightly linked to the host diet, we did not fully explore potential relationship and association with additional confounding factors, such as age, BMI, food diversity, and overall industrialized or non-industrialized food consumption, mostly due to lack of available shotgun sequencing data from DFs intervention trials. Second, our pipeline and the pertaining enzyme, gene, and fiber catalogs were obtained by manually searching, curating, and annotating available data, and following a relatively naïve approach. Accordingly, for example, our current fiber catalog does not cover all known DFs, partly due to the complex definition of DFs in the literature. We also did not consider or handle different polymerization degrees of DFs or enzymes that are integral members of the fiber degradation pathway, such as phosphatases, esterases, transporters, and glycan-binding proteins. Adding such elements in the future may improve our framework’s ability and resolution in describing the host fiber degradation profile, but may also increase its complexity, making it more challenging to draw clear insights and to interpret obtained profiles. Finally, in its current form, our framework handles only whole-shotgun sequencing data and not 16S amplicon sequencing. In principle, functional profiles, and specifically EC abundances, can be inferred from 16S data using PICRUSt [58] and then incorporated into our framework. Yet, given the potential inaccuracy in such mapping, in this first proof-of-concept work, we opted for a simpler and more straightforward model, focusing on a prominent set of dietary fibers and their degradation as inferred directly from high-resolution shotgun data.

## Conclusions

Our framework sheds new light not only on the microbiome’s ability to degrade complex carbohydrates in the form of DFs, but also on our ability to extract such information from metagenomics data. We show the importance of treating DFs as distinct entities, offering novel insights into the connection between microbiome and dietary habits. It is our hope that this framework will serve as a stepping stone toward additional in-depth analyses of the microbiome, DF consumption, and their interactions.

## Methods

### Shotgun metagenomics data

Metagenomic samples were downloaded from the ENA database using the following project accession numbers PRJNA552163 (mice, *n*=15), PRJNA39711 (primates, *n*=89), PRJNA392180 (Hadza, *n*=35), PRJNA268964 (Peru, *n*=36), PRJEB7774 (Austria, *n*=33), PRJNA422434 (China, *n*=144), PRJEB5224 (Spain, *n*=109), PRJNA397112 (India, *n*=46), PRJNA328899 (Mongolia, *n*=107), PRJNA319574 (Netherlands, *n*=150), SRR8791405 (Ghana, *n*=40), PRJNA529400 (Tanzania, *n*=55), and PRJNA504891 (Ethiopia, *n*=40). All samples were processed in the same manner. We subsampled each sample to a depth of 4 × 10^6^ sequences using seqtk and discarded samples with sequencing depth below this threshold. Five samples were discarded from the primate’s dataset, as they were obtained from a grass-eating animal (*Theropithecus gelada*) and not from folivore/non-folivore primates.

*Prevotella copri* genomes were downloaded directly from the Segata lab’s website (http://segatalab.cibio.unitn.it/data/Pcopri_Tett_et_al.html).

### Taxonomic annotation

MetaPhlAn2 [59] was used to obtain genus and species taxonomic profiles. Bacterial taxa whose abundance was < 0.01% in > 90% of the samples were filtered out.

### Fiber selection and structures, enzyme annotation, and enzyme-fiber interaction matrix

DFs were selected according to the metacyc compound annotation system. Specifically, we obtained all dietary fibers that were classified under polysaccharides in the annotation system and that their structure could be derived distinctly using metacyc (as some DFs’ structure was not detailed or was ambiguous).

Following DF selection, we carefully annotated the set of chemical bonds that are present in each DF, according to the structure described by metacyc. DFs with the same higher classification but different bonds were categorized under the same annotation. For example, galactooligosaccharides (GOS) with b1-3, b1-4, or b1-6 bonds were all categorized under GOS with no distinction between them.

The annotation of enzymes relied heavily upon the GlyDeR annotation system [34] and was extended to include all GH and PL enzymes, as denoted by their EC number (3.2.1.*, 4.2.2.*).

Combining the manual curation of both enzymes and DFs, we constructed an enzyme-fiber interaction matrix, in which each entry *M*_*i,j*_ denotes whether enzyme *i* has the capacity to break down a bond present in dietary fiber *j*.

### Building functional databases

We downloaded all GH and PL protein sequences from the UniProt database, denoting the 3.2.1.* and 4.2.2.* EC numbers, using both Swiss-prot sequences (manually annotated and reviewed sequences) and Trembl sequences (automatically annotated). We then used their amino acid sequences to build a reference database of all the fiber-degrading enzymes using Diamond. We also downloaded all the protein sequences with an annotation to an EC number and built an additional database using Diamond which was used to obtain the *functional complete profile*.

### Creating functional profiles

In order to generate the functional profiles described in this paper (FCP and FFP), we mapped the subsampled metagenomic samples to the functional databases described above. Diamond translated search was used to align the metagenomic reads to each database, and the best match detected was selected with default parameters (i.e., *e*-value < 10). We grouped the matched proteins, detected by diamond, by their EC annotation to create a vector of enzymes’ counts for each sample. If a single protein sequence had more than one annotation for an EC, all hits associated with it were discarded to avoid ambiguity, resulting in a total of ~1% of the hits lost.

In addition, we have performed a false discovery rate estimation for our EC mapping, using a simulation-based analysis. Specifically, we downloaded 30 prominent gut bacterial genomes and identified all the GH and PL enzymes in their genomes. These identified sequences were then masked from the original genomes, which were used to simulate 10M shotgun metagenomic reads using InSilicoSeq [60] and Hiseq as the error model. Mapping the obtained 10M reads to our GH and PL curated database has resulted in only 216 hits. In contrast, performing the same simulation analysis without masking GH and PL enzymes resulted in 186,610 hits. This suggests that our mapping is very specific with a negligible false discovery rate of ~0.1%.

Finally, similar to the taxonomic annotation, we discarded enzymes whose abundances were lower than 0.01% of the overall sample abundance in 90% of the samples to receive the final functional profiles (FCP and FFP). To generate the IFDP, we multiplied the filtered FFP matrix (with samples as rows and enzymes as columns) by the enzyme-fiber interaction matrix (enzymes as rows and DFs as columns), resulting in an aggregation of hits for all the enzymes that can breakdown, degrade, or ferment part of the DF for all the DFs.

### Abundance normalization using MUSICC

Metagenomes were normalized using MUSICC [61]. To this end, we obtained the sequences of 76 universally single copy genes from the original paper [61] and built a database using Diamond. For each sample, we mapped the entire metagenome content to this database, with the same parameters we used for the creation of the IFDP. We used the median of the counts of the universally single copy mapped genes as the normalization variable. Next, we obtained the counts of PL and GH enzymes in each metagenome, corrected them for the length of the genes, computed the relative abundance of each gene, and divided by the normalization variable calculated above. The obtained profile was then multiplied by the enzyme-fiber interaction matrix (Fig. 1C), resulting in the MUSICC-corrected IFDP.

### Statistical analysis

To quantitatively compare our predictions to the results of the in vitro bacterial growth assay, we recorded all cases in which this assay suggested that a certain clade can grow on a given fiber (“+” in Table S3 in ref. [37]), and all the cases in which this assay suggested that it cannot grow (“-” in the same table). We similarly ranked the clades according to their inferred ability to degrade each fiber as suggested by the IFDP. We then compared the observed capacity from the in vitro assay to the ranked inferred degradation capacity, measuring the agreement between lack of growth capacity in the assay and low IFDP scores (lowest or second lowest score for that fiber) and likewise the agreement between growth capacity in the assay and high IFDP scores (highest or second highest score for that fiber). To determine the significance of the calculated agreement, we shuffled the IFDP scores for each fiber among the different clades 2000 times, repeated the analysis above, and quantified the probability of observing such agreement by chance.

For the analysis of the primate dataset, we used PERMANOVA with two different stratifications for the randomization of labels (a technique often described as cluster sampling). For the first stratification, we switched labels among all the samples, considering the species of the host and allocating all the samples from this species to the same label. For the second stratification, we stratified samples based on their phylogeny class. We calculated the PERMANOVA statistics on the Euclidean distances of the principal components for the various profiles.

For the Hadza dataset analysis, a random forest classifier was trained for 500 iterations. In each iteration, the data was split into a train and a validation set, with 40% of the data used as the validation set, balanced among the two groups. Default parameters were used for the model with a different random seed for each iteration.

The Mann-Whitney *U* test was used for all univariate testing. All *p* values displayed in the text are corrected for multiple hypotheses.

Code for statistical testing and figure generation was written in python. Skbio was used to calculate alpha diversity; scipy was used for statistical testing; sklearn was used to implement and test random forest models, PCA, and distance calculations; matplotlib, seaborn, and add_stat_anot were used to generate plots; and pandas and numpy were used for data manipulations.

## Supplementary Information

**Additional file 1: Supplementary Table S1.** Annotation of DFs to the set of bonds they contain.

**Additional file 2: Supplementary Table S2.** Annotation of enzymes to the set of bonds they break down.

**Additional file 3: Figure S1.** Validation analysis of the 1,100 *Prevotella copri* genomes: **(A)** Hierarchical clustering of the IFDP of 1,100 *Prevotella copri* genomes. **(B-C)** Principal component analysis of 1,100 *Prevotella copri* genomes as represented by (B) their IFDP or (C) their FFP. **(D)** Scatter plot describing the correlation between the first principal components obtained in the principal component analyses described in panels B and C. Labeled colors on the top in panel A and of dots in panels B and C correspond to the clade of each genome as identified by Tett *et al.* [36].

**Additional file 4: Figure S2.** Bar plots of the degradation capacity of specific DFs of interest in various datasets. **(A-D)** Bar plots describing the inferred microbiome’s degradation capacity of (A) cellulose, (B) xyloglucan, (C) inulin, and (D) Levan, in folivore vs. non-folivore primates. **(E-H)** Bar plots describing the inferred microbiome’s degradation capacity of (E) inulin, (F) pectin, (G) arabinan, and (H) rhamnogalacturonan, in the Hadza tribe in the wet vs. dry seasons. **(I-M)** Bar plots describing the inferred microbiome’s degradation capacity of (I) arabinoxylan, (J) xylan, (K) pectin, (L) resistant starch, and (M) carrageenan, in the Matses and Tunapuco Peruvian tribes. *: *p*<0.05, **: *p*<0.01, ***: *p*<0.001, ****: *p*<0.0001. *P*-values are corrected for multiple hypotheses.

**Additional file 5: Figure S3.** Heat map showing the correlations between the first principal component of each profile. Color bar denotes the spearman r in absolute value.

**Additional file 6: Figure S4.** Alpha diversity of rural and industrialized populations. **(A-C)** Box plots of the Shannon diversity index computed on (A) FFP, (B) FCP, and (C) IFDP, comparing the diversity in the Matses and Tunapuco tribes and in an industrialized cohort from the same study. ***: *p*<0.001, ****: *p*<0.0001.

## Data Availability

All data generated or analyzed during this study are included in this published article, its supplementary information files, and publicly available repositories. Code and required datasets for processing metagenomic data and generating Inferred Fiber Degradation Profiles (IFDPs), along with a walkthrough and step-by-step tutorial are available at https://github.com/borenstein-lab/IFDP.

## References

[1] Nicholson JK, Holmes E, Kinross J, Burcelin R, Gibson G, Jia W, et al. Host-gut microbiota metabolic interactions. Science (80- ). 2012. 10.1126/science.1223813. 10.1126/science.1223813 22674330

[2] David LA, Maurice CF, Carmody RN, Gootenberg DB, Button JE, Wolfe BE, et al. Diet rapidly and reproducibly alters the human gut microbiome. Nature. 2014. 10.1038/nature12820. 10.1038/nature12820 PMC3957428 24336217

[3] Kolodziejczyk AA, Zheng D, Elinav E. Diet–microbiota interactions and personalized nutrition. Nat Rev Microbiol. 2019. 10.1038/s41579-019-0256-8. 10.1038/s41579-019-0256-8 31541197

[4] Koropatkin NM, Cameron EA, Martens EC. How glycan metabolism shapes the human gut microbiota. Nat Rev Microbiol. 2012. 10.1038/nrmicro2746. 10.1038/nrmicro2746 PMC4005082 22491358

[5] Johnson AJ, Vangay P, Al-Ghalith GA, Hillmann BM, Ward TL, Shields-Cutler RR, et al. Daily sampling reveals personalized diet-microbiome associations in humans. Cell Host Microbe. 2019. 10.1016/j.chom.2019.05.005. 10.1016/j.chom.2019.05.005 31194939

[6] De Angelis M, Ferrocino I, Calabrese FM, De Filippis F, Cavallo N, Siragusa S, et al. Diet influences the functions of the human intestinal microbiome. Sci Rep. 2020. 10.1038/s41598-020-61192-y. 10.1038/s41598-020-61192-y PMC7060259 32144387

[7] Sonnenburg JL, Bäckhed F. Diet–microbiota interactions as moderators of human metabolism. Nature. 2016;535:56–4. 10.1038/nature18846. 10.1038/nature18846 PMC5991619 27383980

[8] Porter NT, Martens EC. The critical roles of polysaccharides in gut microbial ecology and physiology. Annu Rev Microbiol. 2017. 10.1146/annurev-micro-102215-095316. 10.1146/annurev-micro-102215-095316 28657886

[9] Desai MS, Seekatz AM, Koropatkin NM, Kamada N, Hickey CA, Wolter M, et al. A Dietary fiber-deprived gut microbiota degrades the colonic mucus barrier and enhances pathogen susceptibility. Cell. 2016. 10.1016/j.cell.2016.10.043. 10.1016/j.cell.2016.10.043 PMC5131798 27863247

[10] Menni C, Jackson MA, Pallister T, Steves CJ, Spector TD, Valdes AM. Gut microbiome diversity and high-fibre intake are related to lower long-term weight gain. Int J Obes (Lond). 2017. 10.1038/ijo.2017.66. 10.1038/ijo.2017.66 PMC5500185 28286339

[11] Koh A, De Vadder F, Kovatcheva-Datchary P, Bäckhed F. From dietary fiber to host physiology: short-chain fatty acids as key bacterial metabolites. Cell. 2016. 10.1016/j.cell.2016.05.041. 10.1016/j.cell.2016.05.041 27259147

[12] Patnode ML, Beller ZW, Han ND, Cheng J, Peters SL, Terrapon N, et al. Interspecies competition impacts targeted manipulation of human gut bacteria by fiber-derived glycans. Cell. 2019. 10.1016/j.cell.2019.08.011. 10.1016/j.cell.2019.08.011 PMC6760872 31539500

[13] Chen T, Long W, Zhang C, Liu S, Zhao L, Hamaker BR. Fiber-utilizing capacity varies in Prevotella- versus Bacteroides-dominated gut microbiota. Sci Rep. 2017. 10.1038/s41598-017-02995-4. 10.1038/s41598-017-02995-4 PMC5453967 28572676

[14] Hughes SA, Shewry PR, Gibson GR, McCleary BV, Rastall RA. In vitro fermentation of oat and barley derived β-glucans by human faecal microbiota. FEMS Microbiol Ecol. 2008. 10.1111/j.1574-6941.2008.00478.x. 10.1111/j.1574-6941.2008.00478.x 18430007

[15] Li W, Wang K, Sun Y, Ye H, Hu B, Zeng X. Influences of structures of galactooligosaccharides and fructooligosaccharides on the fermentation in vitro by human intestinal microbiota. J Funct Foods. 2015. 10.1016/j.jff.2014.12.044.

[16] Bang SJ, Kim G, Lim MY, Song EJ, Jung DH, Kum JS, et al. The influence of in vitro pectin fermentation on the human fecal microbiome. AMB Express. 2018. 10.1186/s13568-018-0629-9. 10.1186/s13568-018-0629-9 PMC6004267 29909506

[17] Sunvold GD, Hussein HS, Fahey GC, Merchen NR, Reinhart GA. In vitro fermentation of cellulose, beet pulp, citrus pulp, and citrus pectin using fecal inoculum from cats, dogs, horses, humans, and pigs and ruminal fluid from cattle. J Anim Sci. 1995. 10.2527/1995.73123639x. 10.2527/1995.73123639x 8655439

[18] Gurry T, Dannenberg PH, Finlayson SG, Hughes TK, Macias-Trevino C, Owusu-Boaitey K, et al. Predictability and persistence of prebiotic dietary supplementation in a healthy human cohort. Sci Rep. 2018. 10.1038/s41598-018-30783-1. 10.1038/s41598-018-30783-1 PMC6107591 30139999

[19] Makki K, Deehan EC, Walter J, Bäckhed F. The impact of dietary fiber on gut microbiota in host health and disease. Cell Host Microbe. 2018. 10.1016/j.chom.2018.05.012. 10.1016/j.chom.2018.05.012 29902436

[20] Davis LMG, Martínez I, Walter J, Goin C, Hutkins RW. Barcoded pyrosequencing reveals that consumption of galactooligosaccharides results in a highly specific bifidogenic response in humans. PLoS One. 2011. 10.1371/journal.pone.0025200. 10.1371/journal.pone.0025200 PMC3180383 21966454

[21] Holscher HD, Bauer LL, Gourineni V, Pelkman CL, Fahey GC, Swanson KS. Agave inulin supplementation affects the fecal microbiota of healthy adults participating in a randomized, double-blind, placebo-controlled, crossover trial. J Nutr. 2015. 10.3945/jn.115.217331. 10.3945/jn.115.217331 26203099

[22] Deehan EC, Yang C, Perez-Muñoz ME, Nguyen NK, Cheng CC, Triador L, et al. Precision microbiome modulation with discrete dietary fiber structures directs short-chain fatty acid production. Cell Host Microbe. 2020. 10.1016/j.chom.2020.01.006. 10.1016/j.chom.2020.01.006 32004499

[23] Tandon D, Haque MM, Gote M, Jain M, Bhaduri A, Dubey AK, et al. A prospective randomized, double-blind, placebo-controlled, dose-response relationship study to investigate efficacy of fructo-oligosaccharides (FOS) on human gut microflora. Sci Rep. 2019. 10.1038/s41598-019-41837-3. 10.1038/s41598-019-41837-3 PMC6445088 30940833

[24] Zhao L, Zhang F, Ding X, Wu G, Lam YY, Wang X, et al. Gut bacteria selectively promoted by dietary fibers alleviate type 2 diabetes. Science (80- ). 2018. 10.1126/science.aao5774. 10.1126/science.aao5774 29590046

[25] Trompette A, Gollwitzer ES, Yadava K, Sichelstiel AK, Sprenger N, Ngom-Bru C, et al. Gut microbiota metabolism of dietary fiber influences allergic airway disease and hematopoiesis. Nat Med. 2014. 10.1038/nm.3444. 10.1038/nm.3444 24390308

[26] Myhrstad MCW, Tunsjø H, Charnock C, Telle-Hansen VH. Dietary fiber, gut microbiota, and metabolic regulation—current status in human randomized trials. Nutrients. 2020. 10.3390/nu12030859. 10.3390/nu12030859 PMC7146107 32210176

[27] Cantu-Jungles TM, Hamaker BR. New view on dietary fiber selection for predictable shifts in gut microbiota. MBio. 2020. 10.1128/mBio.02179-19. 10.1128/mBio.02179-19 PMC7029134 32071263

[28] Lombard V, Bernard T, Rancurel C, Brumer H, Coutinho PM, Henrissat B. A hierarchical classification of polysaccharide lyases for glycogenomics. Biochem J England. 2010;432:437–44. 10.1042/BJ20101185. 10.1042/BJ20101185 20925655

[29] Henrissat B (1991). A classification of glycosyl hydrolases based on amino acid sequence similarities. Biochem J.

[30] El KA, Armougom F, Gordon JI, Raoult D, Henrissat B (2013). The abundance and variety of carbohydrate-active enzymes in the human gut microbiota. Nat Rev Microbiol.

[31] Huang L, Zhang H, Wu P, Entwistle S, Li X, Yohe T, et al. DbCAN-seq: a database of carbohydrate-active enzyme (CAZyme) sequence and annotation. Nucleic Acids Res. 2018. 10.1093/nar/gkx894. 10.1093/nar/gkx894 PMC5753378 30053267

[32] Terrapon N, Lombard V, Drula É, Lapébie P, Al-Masaudi S, Gilbert HJ, et al. PULDB: The expanded database of Polysaccharide Utilization Loci. Nucleic Acids Res. 2018. 10.1093/nar/gkx1022. 10.1093/nar/gkx1022 PMC5753385 29088389

[33] Grondin JM, Tamura K, Déjean G, Abbott DW, Brumer H. Polysaccharide utilization loci: fueling microbial communities. J Bacteriol. 2017. 10.1128/JB.00860-16. 10.1128/JB.00860-16 PMC5512228 28138099

[34] Eilam O, Zarecki R, Oberhardt M, Ursell LK, Kupiec M, Knight R, et al. Glycan degradation (GlyDeR) analysis predicts mammalian gut microbiota abundance and host diet-specific adaptations. MBio. 2014. 10.1128/mBio.01526-14. 10.1128/mBio.01526-14 PMC4145686 25118239

[35] Bateman A. UniProt: a worldwide hub of protein knowledge. Nucleic Acids Res. 2019. 10.1093/nar/gky1049. 10.1093/nar/gky1049 PMC6323992 30395287

[36] Tett A, Huang KD, Asnicar F, Fehlner-Peach H, Pasolli E, Karcher N, et al. The Prevotella copri complex comprises four distinct clades underrepresented in westernized populations. Cell Host Microbe. 2019. 10.1016/j.chom.2019.08.018. 10.1016/j.chom.2019.08.018 PMC6854460 31607556

[37] Fehlner-Peach H, Magnabosco C, Raghavan V, Scher JU, Tett A, Cox LM, et al. Distinct polysaccharide utilization profiles of human intestinal Prevotella copri isolates. Cell Host Microbe. 2019;26:680–90.e5. Available from: https://linkinghub.elsevier.com/retrieve/pii/S1931312819305372. 10.1016/j.chom.2019.10.013. 10.1016/j.chom.2019.10.013 PMC7039456 31726030

[38] Zhang Q, Xiao X, Zheng J, Li M, Yu M, Ping F, et al. Influence of maternal inulin-type prebiotic intervention on glucose metabolism and gut microbiota in the offspring of C57BL mice. Front Endocrinol (Lausanne). 2019. 10.3389/fendo.2019.00675. 10.3389/fendo.2019.00675 PMC6779716 31632351

[39] Amato KR, Sanders JG, Song SJ, Nute M, Metcalf JL, Thompson LR, et al. Evolutionary trends in host physiology outweigh dietary niche in structuring primate gut microbiomes. ISME J. 2019. 10.1038/s41396-018-0175-0. 10.1038/s41396-018-0175-0 PMC6461848 29995839

[40] Smits SA, Leach J, Sonnenburg ED, Gonzalez CG, Lichtman JS, Reid G, et al. Seasonal cycling in the gut microbiome of the Hadza hunter-gatherers of Tanzania. Science (80- ). 2017. 10.1126/science.aan4834. 10.1126/science.aan4834 PMC5891123 28839072

[41] Sfiligoj M, Hribernik S, Stana K, Kree T. Plant fibres for textile and technical applications. Adv Agrophysical Res. 2013. 10.5772/52372.

[42] Hayashi T, Kaida R. Functions of xyloglucan in plant cells. Mol Plant. 2011. 10.1093/mp/ssq063. 10.1093/mp/ssq063 20943810

[43] Marlowe FW, Berbesque JC. Tubers as fallback foods and their impact on Hadza hunter-gatherers. Am J Phys Anthropol. 2009. 10.1002/ajpa.21040. 10.1002/ajpa.21040 19350623

[44] Venter SM, Witkowski ETF. Baobab (Adansonia digitata L.) fruit production in communal and conservation land-use types in Southern Africa. For Ecol. Manage. 2011. 10.1016/j.foreco.2010.11.017.

[45] Li XN, Sun J, Shi H, Yu LL, Ridge CD, Mazzola EP, et al. Profiling hydroxycinnamic acid glycosides, iridoid glycosides, and phenylethanoid glycosides in baobab fruit pulp (Adansonia digitata). Food Res Int. 2017. 10.1016/j.foodres.2017.06.025. 10.1016/j.foodres.2017.06.025 PMC5555379 28784541

[46] Obregon-Tito AJ, Tito RY, Metcalf J, Sankaranarayanan K, Clemente JC, Ursell LK, et al. Subsistence strategies in traditional societies distinguish gut microbiomes. Nat Commun. 2015. 10.1038/ncomms7505. 10.1038/ncomms7505 PMC4386023 25807110

[47] Sadler MJ (2015). Foods, nutrients and food ingredients with authorised EU health claims: volume 2.

[48] Aarnio M, Winter T, Kujala U, Kaprio J. Associations of health related behaviour, social relationships, and health status with persistent physical activity and inactivity: a study of Finnish adolescent twins. Br J Sports Med. 2002. 10.1136/bjsm.36.5.360. 10.1136/bjsm.36.5.360 PMC1724541 12351335

[49] Rauscher-Gabernig E, Mischek D, Moche W, Prean M. Dietary intake of dioxins, furans and dioxin-like PCBs in Austria. Food Addit Contam - Part A Chem Anal Control Expo Risk Assess. 2013. 10.1080/19440049.2013.814169. 10.1080/19440049.2013.814169 23869904

[50] Sustainable food consumption: trends and opportunities. Osterreichische Akademie der Wissenschaften. 2008. In, 1–59. 10.1553/sufotrops1.

[51] Slimani N, Fahey M, Welch A, Wirfält E, Stripp C, Bergström E, et al. Diversity of dietary patterns observed in the European Prospective Investigation into Cancer and Nutrition (EPIC) project. Public Health Nutr. 2002. 10.1079/phn2002407. 10.1079/PHN2002407 12639235

[52] Muthayya S, Sugimoto JD, Montgomery S, Maberly GF. An overview of global rice production, supply, trade, and consumption. Ann N Y Acad Sci. 2014. 10.1111/nyas.12540. 10.1111/nyas.12540 25224455

[53] Zhen L, Ochirbat B, Lv Y, Wei YJ, Liu XL, Chen JQ, et al. Comparing patterns of ecosystem service consumption and perceptions of range management between ethnic herders in Inner Mongolia and Mongolia. Environ Res Lett. 2010.10.1088/1748-9326/5/1/015001.

[54] Samaan RA (2017). Dietary fiber for the prevention of cardiovascular disease: fiber’s interaction between gut micoflora, sugar metabolism, weight control and cardiovascular health.

[55] Liu Y, Heying E, Tanumihardjo SA. History, global distribution, and nutritional importance of citrus fruits. Compr Rev Food Sci Food Saf. 2012. 10.1111/j.1541-4337.2012.00201.x.

[56] Manor O, Borenstein E. Systematic characterization and analysis of the taxonomic drivers of functional shifts in the human microbiome. Cell Host Microbe. 2017. 10.1016/j.chom.2016.12.014. 10.1016/j.chom.2016.12.014 PMC5316541 28111203

[57] Eng A, Borenstein E (2018). Taxa-function robustness in microbial communities. Microbiome..

[58] Langille MGI, Zaneveld J, Caporaso JG, McDonald D, Knights D, Reyes JA (2013). Predictive functional profiling of microbial communities using 16S rRNA marker gene sequences. Nat Biotechnol.

[59] Segata N, Waldron L, Ballarini A, Narasimhan V, Jousson O, Huttenhower C. Metagenomic microbial community profiling using unique clade-specific marker genes. Nat Methods. 2012. 10.1038/nmeth.2066. 10.1038/nmeth.2066 PMC3443552 22688413

[60] Gourlé H, Karlsson-Lindsjö O, Hayer J, Bongcam-Rudloff E (2019). Simulating Illumina metagenomic data with InSilicoSeq. Hancock J, editor. Bioinformatics.

[61] Manor O, Borenstein E. MUSiCC: a marker genes based framework for metagenomic normalization and accurate profiling of gene abundances in the microbiome. Genome Biol. 2015. 10.1186/s13059-015-0610-8. 10.1186/s13059-015-0610-8 PMC4391136 25885687

