# Supplementary figures and images for: The microbiome’s fiber degradation profile and its relationship with the host diet
Source: BMC Biol. 2022 Dec 5;20:266. doi: 10.1186/s12915-022-01461-6 (PMC9721016; doi:10.1186/s12915-022-01461-6)

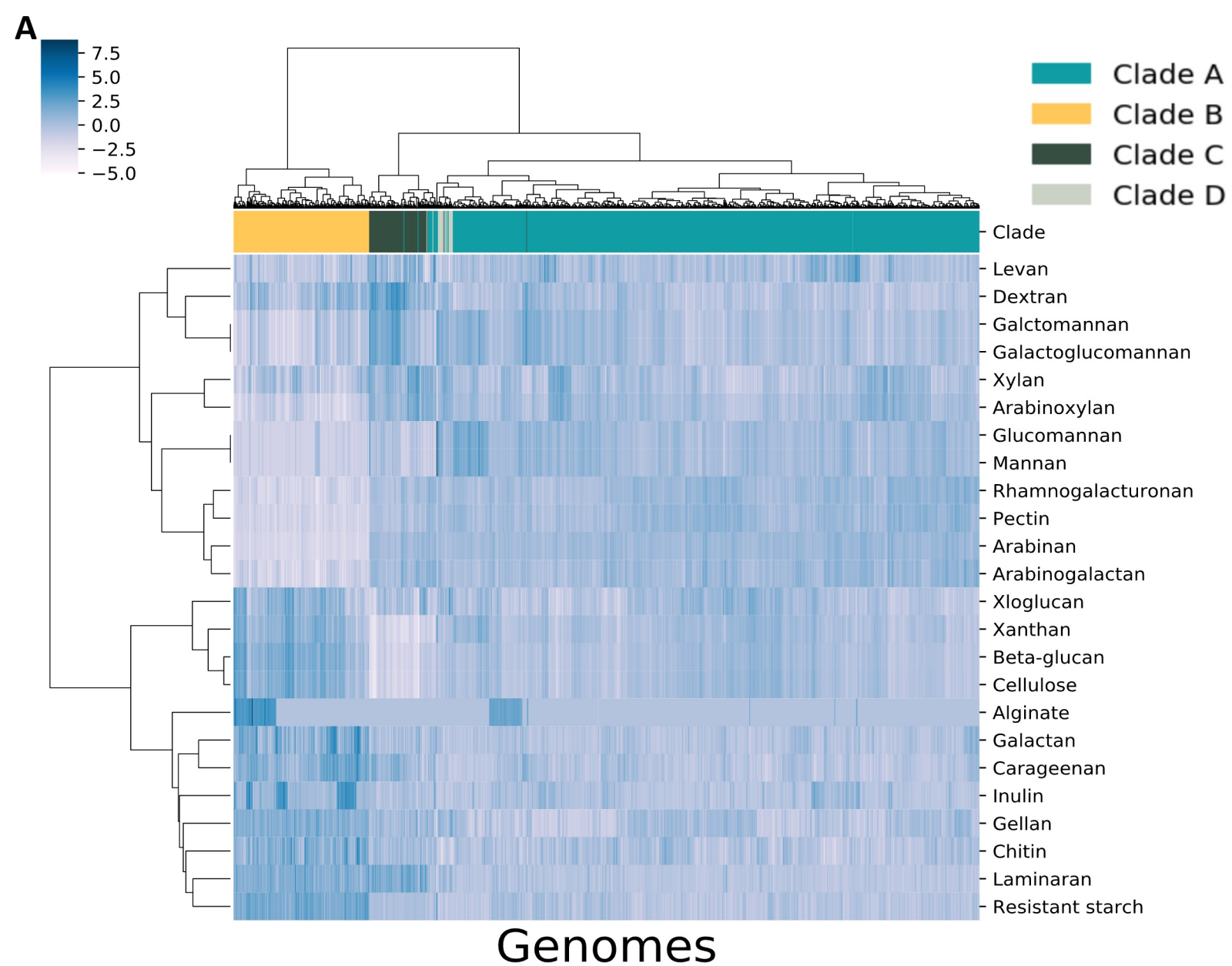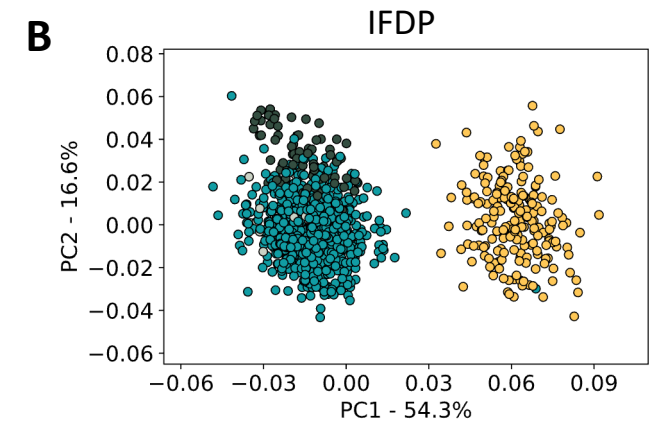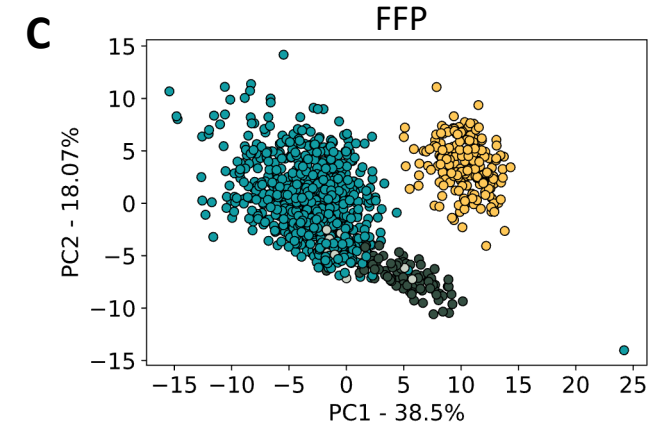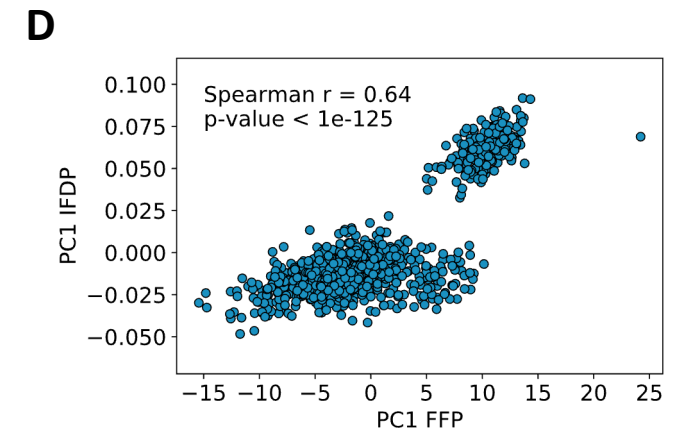

**Figure S1**

Supplement: Supplementary file 3 — Additional file 3: Figure S1. Validation analysis of the 1,100 Prevotella copri genomes: (A) Hierarchical clustering of the IFDP of 1,100 Prevotella copri genomes. (B-C) Principal component analysis of 1,100 Prevotella copri genomes as represented by (B) their IFDP or (C) their FFP. (D) Scatter plot describing the correlation between the first principal components obtained in the principal component analyses described in panels B and C. Labeled colors on the top in panel A and of dots in panels B and C correspond to the clade of each genome as identified by Tett et al. [36]. [file 12915_2022_1461_MOESM3_ESM.pdf]

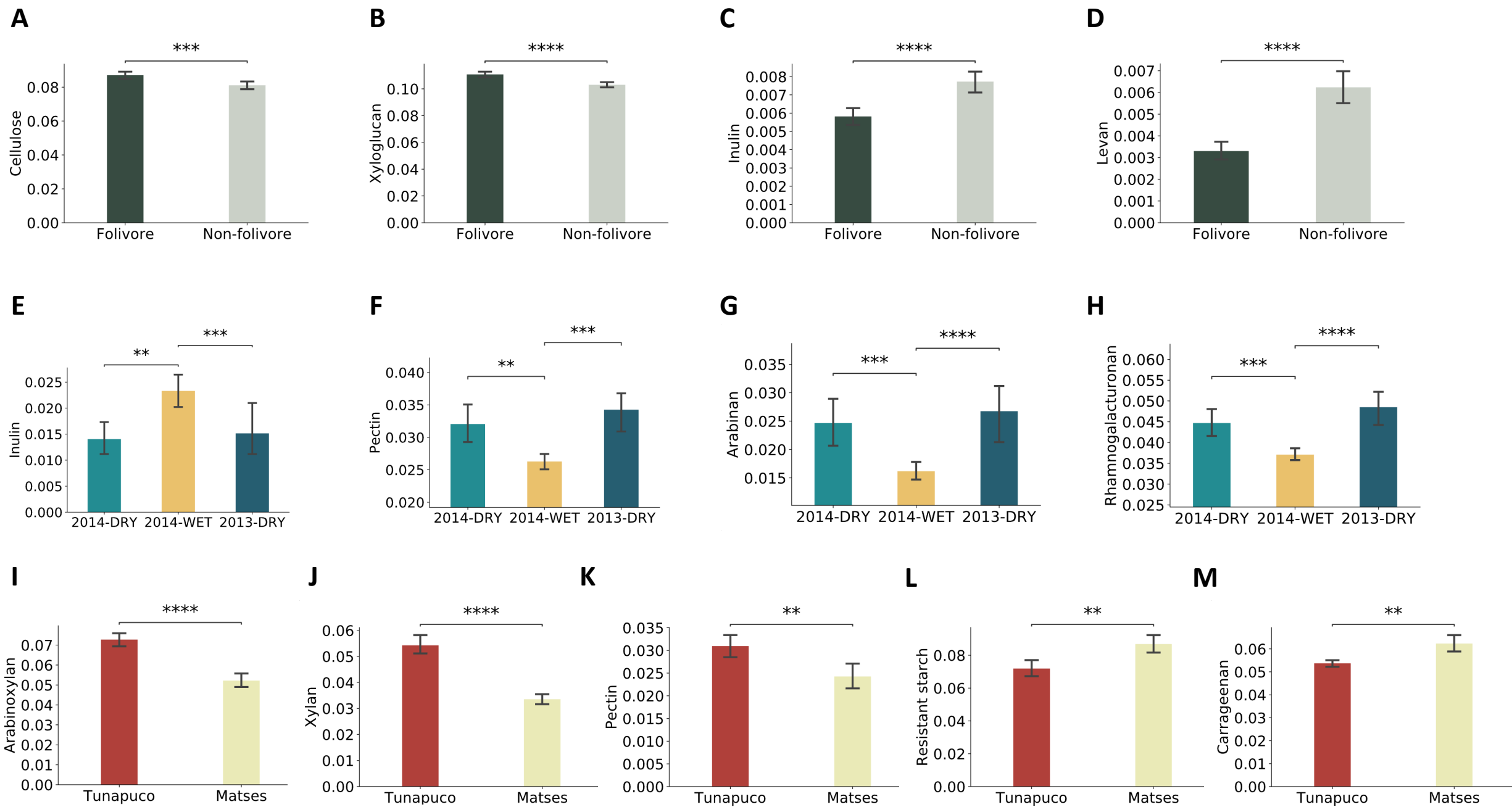

**Figure S2**

Supplement: Supplementary file 4 — Additional file 4: Figure S2. Bar plots of the degradation capacity of specific DFs of interest in various datasets. (A-D) Bar plots describing the inferred microbiome’s degradation capacity of (A) cellulose, (B) xyloglucan, (C) inulin, and (D) Levan, in folivore vs. non-folivore primates. (E-H) Bar plots describing the inferred microbiome’s degradation capacity of (E) inulin, (F) pectin, (G) arabinan, and (H) rhamnogalacturonan, in the Hadza tribe in the wet vs. dry seasons. (I-M) Bar plots describing the inferred microbiome’s degradation capacity of (I) arabinoxylan, (J) xylan, (K) pectin, (L) resistant starch, and (M) carrageenan, in the Matses and Tunapuco Peruvian tribes. *: p<0.05, **: p<0.01, ***: p<0.001, ****: p<0.0001. P-values are corrected for multiple hypotheses. [file 12915_2022_1461_MOESM4_ESM.pdf]

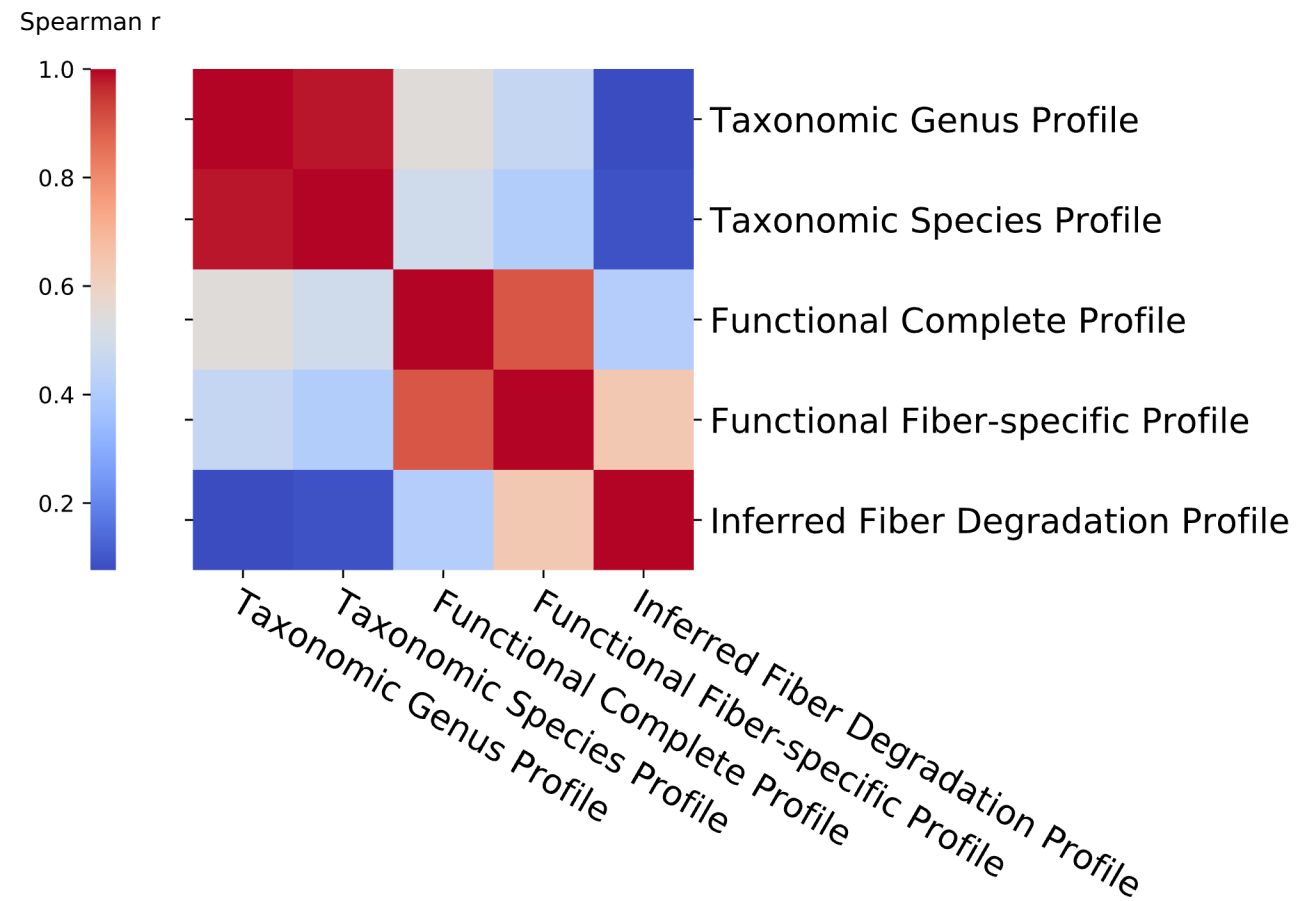

Figure S3

Supplement: Supplementary file 5 — Additional file 5: Figure S3. Heat map showing the correlations between the first principal component of each profile. Color bar denotes the spearman r in absolute value. [file 12915_2022_1461_MOESM5_ESM.pdf]

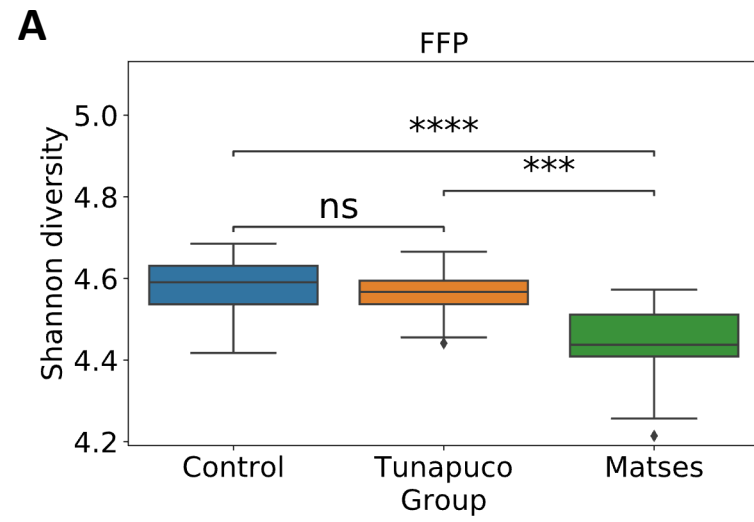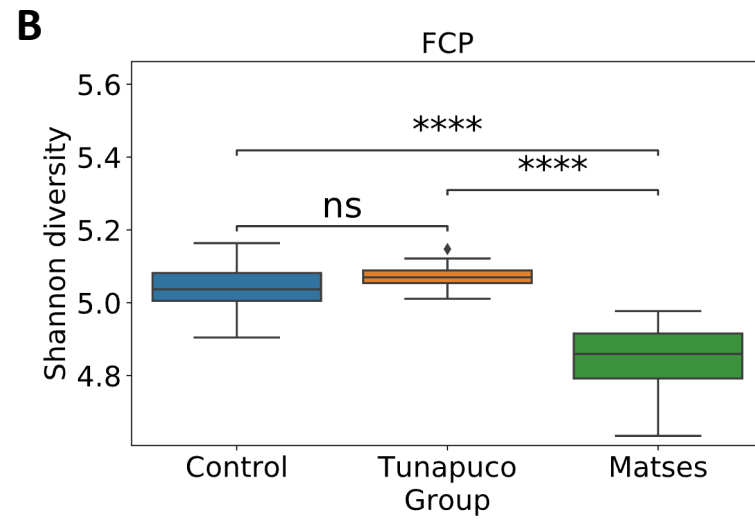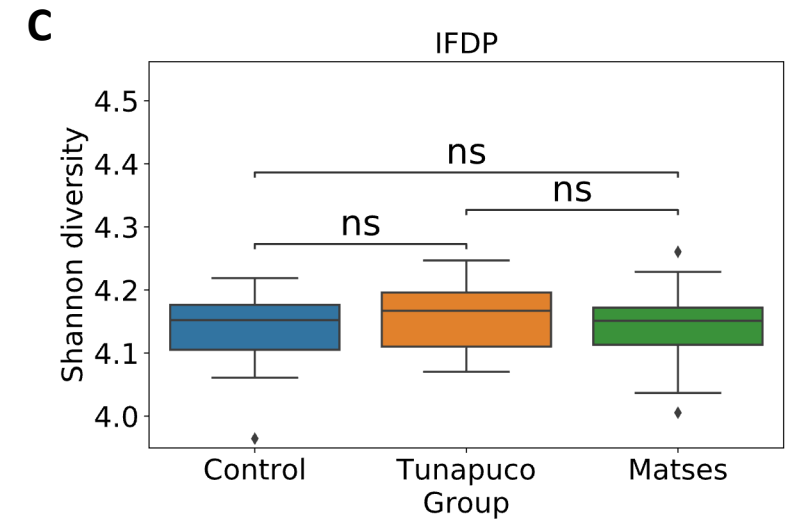

**Figure S4**

Supplement: Supplementary file 6 — Additional file 6: Figure S4. Alpha diversity of rural and industrialized populations. (A-C) Box plots of the Shannon diversity index computed on (A) FFP, (B) FCP, and (C) IFDP, comparing the diversity in the Matses and Tunapuco tribes and in an industrialized cohort from the same study. ***: p<0.001, ****: p<0.0001. [file 12915_2022_1461_MOESM6_ESM.pdf]
